# Exacerbated conceptus signaling does not favor establishment of pregnancy in beef cattle

**DOI:** 10.1186/s40104-018-0302-9

**Published:** 2018-12-07

**Authors:** T. Martins, M. Sponchiado, O. A. Ojeda-Rojas, A. M. Gonella-Diaza, E. O. S. Batista, B. O. Cardoso, C. C. Rocha, A. C. Basso, M. Binelli

**Affiliations:** 1 Department of Animal Reproduction, School of Veterinary Medicine and Animal Science, University of São Paulo, Pirassununga, São Paulo, Brazil; 2 Department of Nutrition and Animal Production, School of Veterinary Medicine and Animal Science, University of São Paulo, Pirassununga, São Paulo, Brazil; 3 In Vitro Brasil, Mogi Mirim, São Paulo, Brazil; 4 Department of Animal Sciences, University of Florida, Gainesville, Florida, USA

**Keywords:** Corpus luteum, Luteolysis, Pregnancy, Uterus

## Abstract

**Background:**

Insufficient production of anti-luteolytic signals by the pre-attachment embryo is considered a major cause of pregnancy failure in cattle. We tested the hypothesis that transfer of multiple blastocysts (*n* = 5/recipient) and progesterone (P4) supplementation amplify anti-luteolytic signaling and reduce embryonic losses in beef cattle. Cows detected in estrus (D0; *n* = 104) were assigned randomly to receive 150 mg of injectable long-acting P4 (iP4) or vehicle (non-iP4) on D4 and transcervical transfer of none or five, grade 1, not-frozen, in vitro-produced blastocysts, on D7. Luteal development and time of structural luteolysis were monitored by ultrasonography. Plasma P4 concentrations were determined on D4, D5 and D7, and daily between D14 and D20. Conceptus signaling was monitored by transcript abundance of interferon-stimulated gene 15 (*ISG15)* in peripheral blood mononuclear cells isolated on D14, D16, D18 and D20. Early embryonic mortality (EEM) was defined as the absence of *ISG15* mRNA upregulation over time and/or luteal regression up to D20. Late embryonic mortality (LEM) was defined as the absence of a conceptus with a heartbeat on pregnancy diagnosis at D30 (PD30) after observing upregulation of *ISG15* mRNA and extension of luteal lifespan. Pregnant cows presented conceptuses with heartbeat at PD30.

**Results:**

On D5, iP4-treated cows had P4 concentrations 2.07-fold greater than non-iP4 treated (*P* < 0.001). On D7, P4 concentrations were similar. Pregnant and LEM animals showed a progressive increase in the abundance of *ISG15* from D14 to D20. iP4-treated cows detected pregnant at PD30 had 1.53-fold greater abundance of *ISG15* mRNA between D14 and D20 than non-iP4 treated cows (*P* = 0.05). iP4 doubled the frequency of EEM while it did not affect LEM. At PD30, embryonic survival was 37.0% vs. 55.6% for iP4-treated vs. control cows. Majority of pregnant cows (71%) presented only a single viable embryo.

**Conclusions:**

A substantial proportion of cows had EEM (31%) and LEM (20%) even after transferring multiple blastocysts. This argues that mortality was due to poor uterine receptivity that could not be reversed by supplemental P4 or overcome by transferring multiple blastocysts. Further, a given uterine environment was not necessarily adequate to all embryos.

## Background

During the first three weeks post-insemination, embryonic mortality accounts for up to two-thirds of overall pregnancy losses in cattle [1–3], constituting a major cause of pregnancy failures. Explanation for this massive loss is related to a functional incompetence of the uterus to support embryonic development, embryonic incompetence or both [4]. However, a clear distinction among these possibilities has proven to be both a scientific and practical challenge.

Uterine environment must be capable to support embryonic growth and elongation between d 4 and 16 post-mating [5–7] and subsequently, it must be able to respond to the elongated conceptus-initiated interferon-tau (IFNT) signaling [8], which will prevent luteal regression. Thus, strategies that stimulate embryo receptivity by the uterus may increase probability of gestational success. For example, a more receptive-uterine status can be generated through increasing concentrations of progesterone (P4) during early diestrus. Indeed, increased concentrations of P4 stimulate endometrial secretions [9], accelerate conceptus elongation and subsequent release of IFNT into the uterine lumen [10–12] in cattle. Despite of such clear positive effect on pregnancy establishment, the reported fertility outcome to early diestrus P4 supplementation is variable [13, 14]. Inconsistencies can be associated with the incidence of early luteolysis (i.e., before D16), which impairs the proper cross-talk between the uterus and the conceptus by D16. Early luteolysis is related to the P4–driven impairment of luteal formation [15, 16] and/or the advancement of endometrial PGF2α release [17]. Alternatively, an asynchrony between stimuli provided by the uterine environment and required by the developing embryo [18] can be also an explanation for the variable fertility results.

The competence of an individual embryo to develop, elongate and signal is intrinsically confounded with the ability of the uterus to support embryonic development. Furthermore, embryonic response to a given uterine environment seems to be variable and unpredictable. For example, Betteridge et al. [19] observed striking differences in the size of conceptuses recovered from superovulated donors on D14 (0.226–57 mm) and on D16 (0.232–150 mm) and this was also true for an individual donor (e.g., there was a range of 4–40 mm from one D14 donor). Similarly, Garret et al. [9] verified that conceptuses recovered after natural mating from D14 uteri of P4-treated cows (37.3 ± 14.9 mm) were longer than those recovered from controls (3.8 ± 1.9 mm); however, they noticed a large variability within and between treatments (control: 1–13 mm; P4 treated: 3–119 mm). Other studies have also reported variability on the length of conceptuses recovered on D14 to D16 post-estrus in cattle [12, 20] and sheep [21]. The reason for this variability remains unclear. Possible explanations could be related to the variation in the blastocyst cell number [22] and asynchrony between trophoblast development and the development of the embryonic disk [23]. Multiple embryo transfer can be used as attempt to sort embryo vs. uterine effects [20, 24]. By transferring 3 to 8 high-grade, in vitro produced blastocysts to recipients, Berg et al. [20] estimated that the fraction of pregnancy failure attributed to embryonic incompetence is expected to be minimum, and this would allow an estimation of the fraction of losses associated with an incompetent uterus.

Thus, rationale for the present study was to 1) stimulate uterine functions to support embryo elongation and survival, by treating recipients with supplemental P4, 2) decrease the random chance of an incompetent embryo to fail to develop and elongate by transferring multiple in vitro-produced blastocysts (*n* = 5/recipient) and 3) increase the potency of conceptus signaling by the additive secretory capacity of multiple conceptuses. Combination of these ingredients was expected to increase the intensity of conceptus signaling (i.e., production of IFNT) and pregnancy success. Hypothesis was that P4 supplementation at early diestrus and transfer of multiple blastocysts reduce early (i.e. between D8 and D20) and late (i.e. after D20) embryonic mortality. Embryonic survival and signaling potency around the maternal recognition period were monitored through mRNA levels of interferon-stimulated gene 15 (*ISG15*) in peripheral blood mononuclear cells (PBMCs). A side-effect of P4 supplementation at early diestrus is an increased proportion of cows that undergo early luteolysis. A second hypothesis tested in the present report was that increased potency of conceptus signaling overcomes the iP4 effect to advance luteolysis, thus, reducing the incidence of early luteolysis.

## Methods

### Animals

Cycling, multiparous, non-suckled, Nelore cows (*Bos taurus indicus*; *n* = 50; 569.9 ± 10.1 kg and 6.1 ± 0.3 years old) were used in three replicates performed in the summer of 2016–2017 in the Southern hemisphere at Fernando Costa Campus of the University of São Paulo (Pirassununga, São Paulo, Brazil). Experimental animals had no apparent abnormalities in the reproductive tract and had a CL in at least one of the weekly ultrasound scans. Cows belonged to the breeding herd of the University of São Paulo and conceived and calved yearly. Thus, cows were considered to be of proven fertility. Animals were kept under grazing conditions and supplemented with corn silage, concentrate and minerals to fulfill their maintenance requirements, and water ad libitum. The cows were managed in accordance with the Ethics and Animal Handling Committee of the School of Veterinary Medicine and Animal Science of the University of São Paulo (CEUA-FMVZ/USP, n° 4664220316).

### Experimental design

**Fig. 1:**
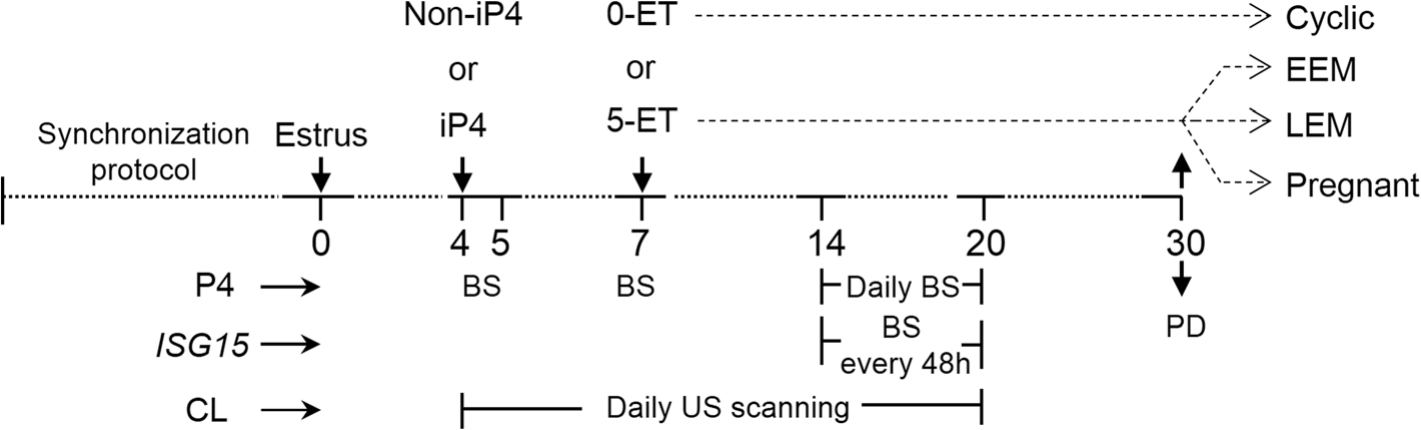
Diagram of the experimental design. Estrous cycle of non-suckled, multiparous Nelore cows was synchronized and estrus was detected (D0; *n* = 104). Animals were assigned randomly to one of the four treatment combinations on a two by two factorial arrangement of the following treatments: vehicle (Non-iP4) or supplementation with 150 mg of injectable long-acting P4 (iP4) on D4 and transfer of none (0-ET) or 5 in vitro-produced blastocysts on D7 (5-ET). Blood samples (BS) were collected on D4, D5, D7 and daily from D14 to D20 to determine circulating P4 concentrations. BS are also collected every 48 h between D14 and D20 for analysis of *ISG15* relative abundance. From D4 to D20, ultrasound (US) scanning was conducted to record total CL area and CL area containing color signals of luteal blood flow for evaluation of CL growth and regression. On D30, pregnancy diagnosis (PD) was based on detection of an embryo with heartbeat at US. Those cows from 5-ET groups were classified retrospectively as experiencing early embryonic mortality (EEM), late embryonic mortality (LEM) or diagnosed as pregnant on D30. Those cows from 0-ET groups were classified as cyclic

Estrous cycles were synchronized using an P4-releasing intravaginal device for 8 d (1.0 g; Sincrogest®, Ourofino Saúde Animal, Cravinhos, SP, Brazil), along with an intramuscular (i.m.) injection of 2 mg of estradiol benzoate (2.0 mL; Sincrodiol®, Ourofino Saúde Animal), followed by an i.m. administration of 0.53 mg of sodium cloprostenol (2.0 mL; PGF2α analogue; Sincrocio®, Ourofino Saúde Animal) given on the day before P4-device removal. Cows were checked for signs of estrus twice a day between 36 and 96 h after P4-releasing device withdrawal with the aid of heat detection patches (Estrotect™; Western Point Inc., Apple Valley, MN). Cows observed in standing estrus and/or presenting an activated heat detection patch were considered in estrus (D0 of study; *n* = 104). Animals were assigned randomly to one of the four treatment combinations on a two-by-two factorial arrangement of the following treatments: vehicle or supplementation with 150 mg of long-acting P4 (iP4, 1.0 mL, i.m., Sincrogest® injectable, Ourofino Saúde Animal) on D4 and transfer of none (0-ET) or 5 in vitro-produced blastocysts on D7 (ET). Thus, the experimental groups were: Non-iP4 + 0-ET (*n* = 24); iP4 + 0-ET (*n* = 26); Non-iP4 + 5-ET (*n* = 27) and iP4 + 5-ET (*n* = 27). This dose was chosen because cycling, multiparous, non-lactating Nelore cows injected with this formulation on D3 after ovulation presented greater plasma P4 concentrations from D3.5–D5.5 than controls [25]. This regime of P4 supplementation has produced both embryotrophic and luteolytic stimuli [25–27]. We used five blastocysts because a greater number of blastocysts could reduce pregnancy rates and growth of conceptuses [20]. The experimental design is illustrated in Fig. 1.

Blood samples were collected via jugular venipuncture into 9 mL heparinized evacuated tubes and placed on ice until centrifugation on D4, D5 and D7 post estrus and daily between D14 and D20 to determine plasma P4 concentrations. Additional blood samples (18 mL) were collected on D14, D16, D18 and D20 for determination of *ISG15* expression in PBMCs. Samples for PBMC isolation were kept at controlled 24 °C until processing on the same day.

### Ultrasound scanning

Ultrasound scanning was performed using a transrectal B-mode ultrasonography apparatus (Mindray M5 equipped with multifrequency linear transducer set to 7.5 MHz) starting when estrus was detected and repeated every 12 h until ovulation. To evaluate the CL development and regression, the ovaries were scanned between D4 and D20 via transrectal B-mode and pulse-wave color Doppler ultrasonography. Total CL area and CL area containing color signals of luteal blood flow (CLBF) were also measured. To determine the maximum CL area, the images were recorded in B-mode as film clips and measured a posteriori, using a tracing function. In CLs having an anechoic fluid-filled cavity, the area of the cavity was subtracted from the total area [28]. Percentage of CL area with CLBF was estimated visually during CL scanning using the same criteria as described previously [25, 29]. Accordingly, a scale from 0 to 100%, with 5 interval points was used for determination of the percentage of the luteal area with CLBF. All scans were performed at a constant color-gain setting (6.5 MHz, Gain: 62 and Pulse Repeated Frequency: 5.3 kHz) and a velocity setting of 5.4 cm/s. Day of structural luteolysis was defined as the day between D11 and D20 when the maximum CL area (cm^2^) and the luteal blood flow decreased by 25% and 50%, respectively, from the respective mean values recorded on D10 and D11, based on previous reports [25, 29].

Pregnancy status for each animal was determined by detection of at least one conceptus with heartbeat using transrectal B-mode ultrasonography on D30. Numbers of viable conceptuses (i.e. with heartbeat) were counted during pregnancy diagnosis. After pregnancy diagnosis on D30, all cows received two injections of PGF2α analogue, 24 h apart to induce abortion. Cows were not submitted to any manipulations for 30 d. The 30–day recovery period is sufficient to allow a proper involution of the previously pregnant reproductive tract [30]. Adequate status of ovaries and uterus were confirmed at the beginning of each estrous synchronization protocol by ultrasound exam. The cows used in the second and third replicates were divided evenly among the four treatments. Across replicates, there was no allocation of a given animal to a previous treatment.

### Embryo transfer procedure

In vitro-produced blastocysts were obtained from a commercial supplier. Cumulus oocyte complexes used to produce blastocysts were aspirated from genetically undefined, predominately *Bos taurus indicus* ovaries obtained from a local slaughterhouse. Embryos were produced according to a standard protocol for in vitro embryo production [31], using frozen-thawed spermatozoa from a single Nelore bull. Five fresh, grade 1 blastocysts, produced in vitro (i.e., not frozen) were placed in straws containing holding medium and transported to our laboratory at 37 °C on D7.

On D7, recipient cows received a caudal epidural anesthesia (2% lidocaine solution; Lidovet®, Bravet, Engenho Novo, RJ, Brazil) immediately before the embryo transfer (ET) procedure. Five in vitro produced blastocysts were placed transcervically in the middle to the cranial third of the uterine horn ipsilateral to the ovary containing the CL, using standard non-surgical technique. For the 0-ET groups, all the procedures were performed but only holding medium was deposited in the uterine lumen.

### Progesterone concentrations measurements

Plasma was collected from blood samples by centrifugation at 2,700 x g for 15 min at 4 °C. Progesterone was assayed by liquid-phase radioimmunoassay (Immuchem™ Double Antibody Progesterone Kit; Cat. 07–170105, MP Biomedicals, NY, USA). The sensitivity of the assay was 0.5 ng/mL. The intra-assay coefficient of variation (CV) for quality control samples was 0.3% (low) and 3.08% (high). The inter-assay CV were 2.48% (low) and 14.24% (high).

Plasma P4 concentrations profiles between D4 and D7 were used to evaluate the effect of iP4 supplementation on circulating P4 levels, and between D14 and D20 to determine the day of functional luteolysis. Functional luteolysis was defined as the day when P4 concentrations decreased > 2 ng/mL between daily samples, followed by a progressive decrease in concentrations to < 1 ng/mL [25].

### Isolation of PBMCs, total RNA extraction and cDNA synthesis

PBMCs were isolated by Ficoll gradient as described by Pugliesi et al. [29]. Briefly, whole blood was mixed with an equal volume of PBS, and the solution was layered onto 15 mL of Ficoll-Paque solution (GE Healthcare), placed in a 50-mL conical tube, and then centrifuged at 1,100 × g for 30 min to obtain the PBMC layer. Mononuclear cells were collected and centrifuged with PBS at 900 × g for 15 min and the contaminating red blood cells were lysed in hypertonic solution for approximately 10 min. Isotonicity was restored by suspending the cells in PBS and centrifuging at 900 × g for 15 min. The resulting pellet was suspended in PBS and the final solution was transferred to a 1.5-mL conical tube. The isolated PBMCs were centrifuged at 3,300 × g for 2 min, the supernatant was removed, and the pellet containing PBMCs was stored in a 1.5-mL conical tube at − 80 °C until RNA extraction.

Total RNA extraction of isolated PBMCs was performed using 1 mL of Trizol™ reagent (Invitrogen, Carlsbad, CA, USA) in accordance to manufacturer’s guidelines. Total RNA extracted was eluted with 40 μL of RNase free water. Concentrations and purity of total RNA in extracts were evaluated using spectrophotometry (NanoVueTM Plus Spectrophotometer, GE Healthcare, UK) by the absorbance at 260 nm and the 260/280 nm ratio, respectively. Absorbance ratios values ranged between 1.7 and 1.9.

Before the reverse transcription, the isolated RNA samples were treated with DNase I (deoxyribonuclease I, Amplification Grade; Invitrogen, Carlsbad, CA, USA) for genomic DNA contamination as per manufacturer’s instructions. Briefly, the treatment with DNase was done at room temperature using 1 μg of total RNA in a 10-μL reaction volume. After 15 min of incubation at room temperature, 1 μL of EDTA (25 mmol/L) was added to stop the enzyme activity and samples were warmed to 65 °C for 10 min. Synthesis of cDNA was performed using the High-Capacity cDNA Reverse Transcription Kit (Life Technologies Corporation, Frederick, MD, USA). A master mix (9 μL) containing random primers, reverse transcriptase enzymes and deoxynucleotides were added to 11 μL of the treated samples. Samples were incubated at 25 °C for 10 min and then at 37 °C for 2 h, subjected to reverse transcriptase inactivation at 85 °C for 5 min, and stored at − 20 °C until PCR analysis.

### Real-time PCR

Analyses of relative abundance of transcripts were performed using Power SYBR Green PCR Master Mix (Life Technologies) for the amplification reactions in a Step One Plus thermocycler (Applied Biosystems Real-Time PCR System; Life Technologies). The primers used for qPCR were obtained from our previous report [32] as follows: 5’-AGAGAGCCTGGCACCAGAAC-3′, forward, and 3’-TTCTGGGCGATGAACTGCTT-5′, reverse, for *ISG15* (NM_174366.1); and 5’-GCCATGGAGCGCTTTGG-3′, forward, and 3’-CCACAGTCAGCAATGGTGATCT-5′, reverse, for *PPIA* (Cyclophilin A; NM_178320.2). Reactions were run in triplicate on 96-well plates sealed with a MicroAmp optical adhesive cover (Life Technologies) using the same qPCR settings as described previously [32]. The raw fluorescence data was extracted from the Step One Plus software with no baseline correction and analyzed using the LinReg PCR software (www.hartfaalcentrum.nl/index.php?main=files&sub=LinRegPCR) for baseline correction and cycle thresholds (Cts) determination, as described by Ruijter et al. [33]. Specifically, the log-linear portion of the amplification curve containing four to six points with the highest *R*^2^ value was considered. Relative transcripts abundance was obtained after normalization of *ISG15* Cts by the endogenous control *PPIA* Ct values, using the equation described by Pfaffl et al. [34].

### Criteria for determining embryo survival or mortality

Cows that received five blastocysts on D7 (5-ET groups) were classified retrospectively as experiencing early embryonic mortality (EEM), late embryonic mortality (LEM) or confirmed pregnant on the day of pregnancy diagnosis (i.e., D30). Cows were included in the EEM group when there was no evidence of embryo presence up to D16, based on the lack of increase in the abundance of *ISG15* mRNA between D14 and D20 and/or the incidence of luteolysis before D20. Conversely, cows classified as LEM presented a clear increase on *ISG15* abundance, a functional CL on D20, but no evidence of a viable embryo on D30. Confirmed pregnant cows and those that received no embryos (0-ET or cyclic group) were used as positive and negative controls, respectively, for comparisons of *ISG15* expression among EEM and LEM groups. These groups are illustrated in Fig. 1.

### Statistical analysis

Data from CL area, CL blood flow, P4 concentrations and *ISG15* expression was analyzed by split-plot ANOVA using the MIXED procedure of SAS (SAS Inst. Inc., Cary, NC, USA) version 9.3. Model included fixed effects of iP4 supplementation, ET treatment, day and their interactions for analysis of CL characteristics and P4 concentrations. For analysis of *ISG15* mRNA relative abundance, the model included fixed effects of iP4 supplementation, group (EEM, LEM, pregnant and cyclic cows), day and their interactions. Random effect of cows nested within treatment combinations was used as the error term. The type of variance–covariance structure used was chosen based on smaller magnitude of the corrected Akaike’s information criterion (AICC). Kenward-Rogers degrees of freedom approximation option of SAS was used to determine the denominator degrees of freedom for tests of fixed effects. The residual and influencing diagnostics outputs from the MIXED procedure were checked for the assumption of normality of the data. Data that were not distributed normally were log transformed before being analyzed statistically. Significance of effects was determined by *F*-test using Type III sums of squares. When treatment by day interactions were significant, the slice command was incorporated into the procedure to determine in which day the treatment effect occurred. When necessary, the DIFF command incorporating the Tukey test correction was applied to evaluate pairwise comparisons among treatment means.

Secondary analyses were performed using only cows submitted to 5-ET aiming to assess the difference in CL features between D4 and D14 and P4 concentrations according to pregnancy status (group). Model included the fixed effects of iP4 supplementation, group, day, and their interactions. To assess the effect of iP4 supplementation on *ISG15* relative abundance, an additional analysis was run considering only cows diagnosed as pregnant. Model included the fixed effects of iP4 supplementation, day, and their interaction.

Variables day to structural/functional luteolysis and day from estrus to P4 < 1.0 ng/mL were analyzed with the MIXED procedure considering fixed effects of iP4 supplementation, ET treatment and their interaction. A new variable was generated by considering the means of P4 concentrations and *ISG15* expression over time to assess effects according to pregnancy status. Model included the fixed effect of group, iP4 supplementation and their interaction. Finally, the effect of iP4 supplementation on the proportion of pregnant cows was assessed by the chi-squared test, using the FREQ procedure of SAS. In all instances, a probability of *P* ≤ 0.05 indicates a significant difference and a probability of 0.05 < *P* ≤ 0.10 indicates a trend toward significance.

## Results

### Animals and groups

The mean ± SEM of the variables size of pre-ovulatory follicle (13.8 ± 0.16 mm) and time from estrus detection to ovulation (25.06 ± 0.61 h) were similar among groups (*P* > 0.10), as expected.

### Effect of P4 supplementation and embryo transfer on ovarian and endocrine variables

**Fig. 2:**
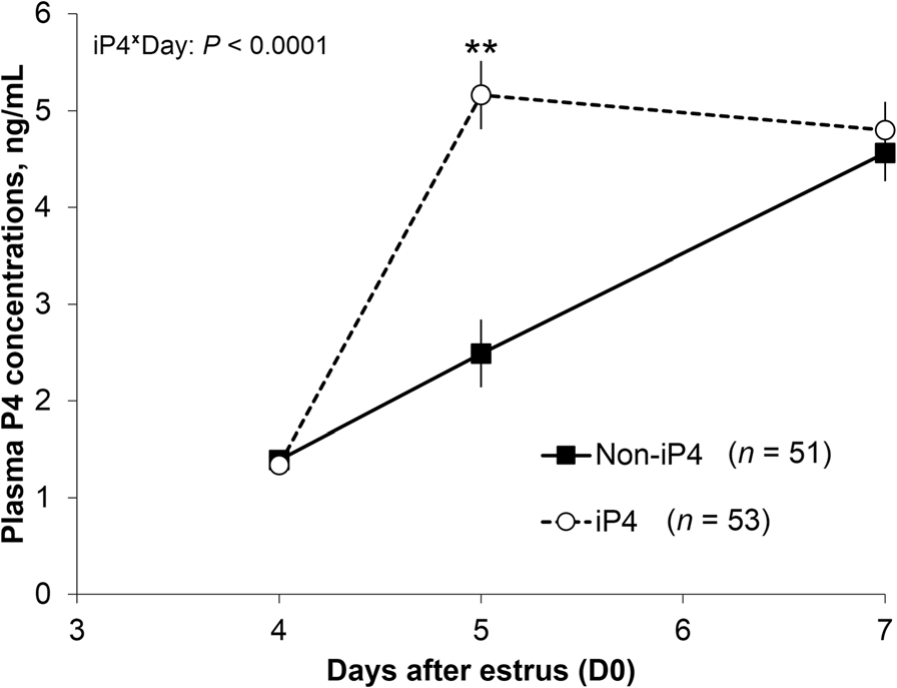
Effect iP4 supplementation on plasma progesterone concentrations. Beef cows detected in estrus (D0) were assigned randomly to receive a single injection of vehicle (Non-iP4) or 150 mg of long acting progesterone (iP4) on D4 and transfer of none or 5 in vitro produced blastocysts on D7. Data were analyzed by split-plot ANOVA and only the significant effect was reported. Data are represented as Least squares means ± SEM. Difference within a day is indicated by asterisks (**P < 0.0001)

Concentrations of P4 in plasma between D4 and D7 were affected by a day by iP4 treatment interaction (Fig. 2). The interaction effect was a consequence of an expected, acute increase (*P* < 0.0001) on average plasma P4 concentrations on D5 in iP4- (5.16 ± 0.35 ng/mL) versus non-iP4-supplemented cows (2.49 ± 0.35 ng/mL). On D7, plasma P4 concentrations between these two groups were similar.

**Fig. 3:**
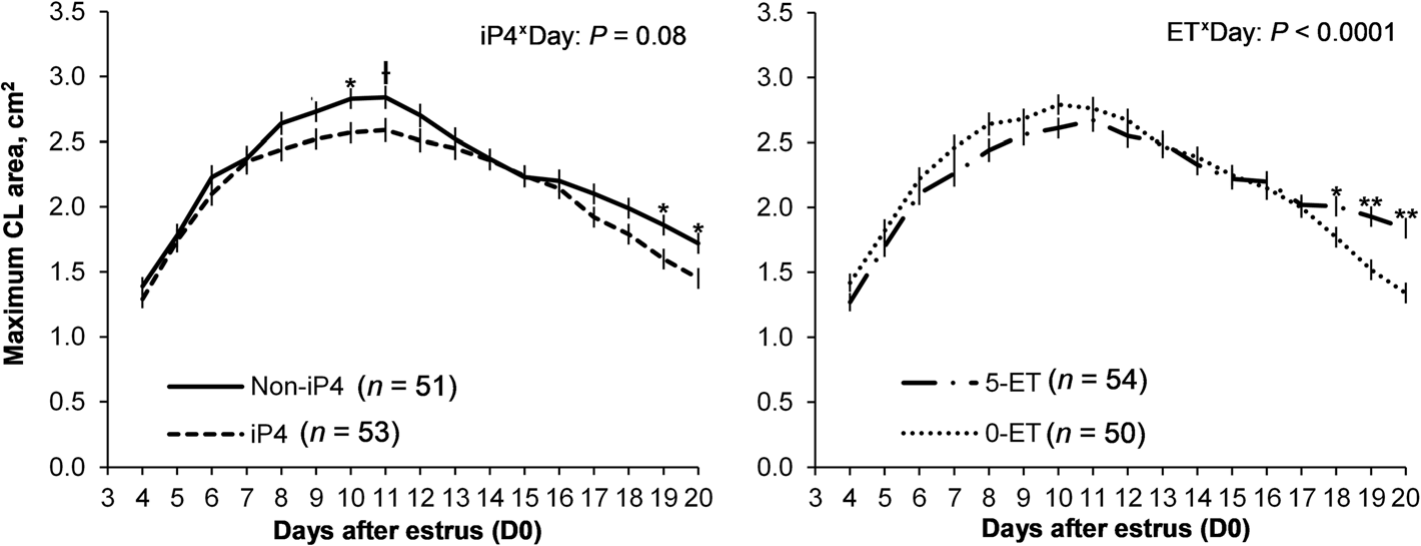
Effect of iP4 supplementation and embryo transfer on the corpus luteum (CL) along diestrus. Beef cows detected in estrus (D0) were assigned randomly to receive a single injection of vehicle (Non-iP4) or 150 mg of long acting progesterone (iP4) on D4 and transfer of none (0-ET) or 5 in vitro produced blastocysts (5-ET) on D7. Maximum area of CL was used for evaluation of CL development and regression. Data were analyzed by split-plot ANOVA and only the significant effects were reported. Data are represented as Least squares means ± SEM. Differences within a day are indicated by asterisks (*P < 0.05 and **P < 0.001) or cross (Ɨ*P* < 0.10)

Treatment with iP4 tended to inhibit CL development (D4 to D11) and hasten CL regression (D14 to D20; day by iP4 treatment interaction; *P* < 0.08; Fig. 3). There was no effect of iP4 on the proportion of luteal area with CLBF nor the concentrations of P4 between D14 and D20 (*P* > 0.10).

Regardless of iP4 supplementation, there was a day by ET treatment interaction effect on CL area (Fig. 3) between D14 and D20. Starting on D18, the average CL area was greater in the 5-ET groups than 0-ET groups. Similarly, from D18 to D20, proportion of luteal area with CLBF and concentrations of P4 was greatest in the 5-ET groups (data no shown). These results reflected a positive effect of the embryo on the maintenance of CL function in cows subjected to the transfer of five blastocysts.

The functional characteristics of CLs and P4 concentrations profiles between D14 to D20 were used to calculate the time of structural and functional luteolysis, respectively (Table 1). Intriguingly, both criteria indicated an approached or significant effect of ET treatment on time of luteolysis, regardless of iP4 supplementation. Time of structural luteolysis in 5-ET cows (16.95 ± 0.39 d) tended to be shorter than 0-ET cows (17.75 ± 0.24 d). Time of functional luteolysis (5-ET: 16.30 ± 0.35 vs. 0-ET: 17.03 ± 0.21 d) and between estrus to P4 < 1.0 ng/mL (5-ET: 16.87 ± 0.39 vs. 0-ET: 17.81 ± 0.23 d) indicated a similar effect. However, iP4 supplementation did not shorten luteal lifespan, despite the impairment of the corpus luteum formation.

**Table 1:** Effect of iP4 supplementation and/or embryo transfer on the CL regression in beef cows

Variable	Non-iP4		iP4		*P* value		
	0-ET (*n* = 24)	5-ET (*n* = 27)	0-ET (*n* = 26)	5-ET (*n* = 27)	iP4	ET	iP4×ET
Number of cows having functional CL on D20^a^	4 (16.7%)	21 (77.8%)	2 (7.7%)	16 (59.3%)	.	.	.
Estrus to structural luteolysis, d^b^	17.58 ± 0.35	17.00 ± 0.63	17.92 ± 0.31	16.91 ± 0.46	0.79	0.09	0.64
Estrus to functional luteolysis, d^c^	17.11 ± 0.31	16.50 ± 0.56	16.96 ± 0.28	16.09 ± 0.41	0.50	0.08	0.75
Estrus to P4 < 1.0 ng/mL, d^d^	17.79 ± 0.35	16.83 ± 0.62	17.83 ± 0.31	16.91 ± 0.46	0.90	0.04	0.97
Number of pregnant cows on D30^e^	.	15 (55.6%)	.	10 (37.0%)	0.16	.	.
Number of non-pregnant cows having functional CL on D30^f^	.	6 (22.2%)	.	5 (18.5%)	0.74	.	.

Beef cows detected in estrus (D0) were assigned randomly to receive a single injection of vehicle (Non-iP4) or 150 mg of long acting progesterone (iP4) on D4 and transfer of none or 5 in vitro-produced blastocysts on D7;

^a^Cows that did not present any of the characteristics described in items 2, 3 and 4, below;

^b^Structural luteolysis was defined as the day between D11 and D21 when the maximum CL area (cm^2^) and the luteal blood flow decreased by 25% and 50%, respectively, from the mean of D10 and D11;

^c^Functional luteolysis was defined as the day when plasma P4 concentrations decreased > 2 ng/mL between samples collected from D14 to D20, and was followed by a progressive decrease in plasma P4 concentrations to < 1 ng/mL;

^d^Day when plasma P4 concentrations first reached < 1.0 ng/mL;

^e,f^Pregnancy status for each animal was determined by detection of at least one conceptus with heartbeat using transrectal B-mode ultrasonography on D30. In non-pregnant cows, the functionality of the CL from D20 was checked according to criteria established for detection of structural luteolysis

### Effect of P4 supplementation on variables related to pregnancy and on the relative abundance of *ISG15*

**Fig. 4:**
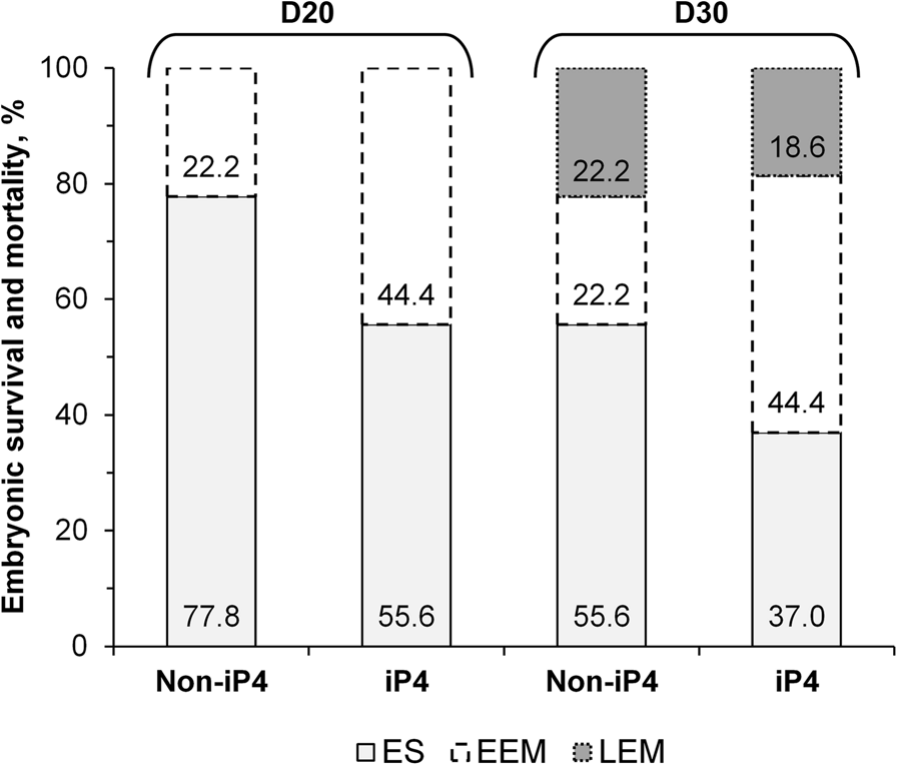
Frequency of pregnancy losses in beef cattle supplemented or not with iP4. Cows detected in estrus (D0) were assigned randomly to receive a single injection of vehicle (Non-iP4; *n* = 27) or 150 mg of long acting progesterone (iP4; *n* = 27) on D4 and transfer of 5 in vitro produced blastocysts on D7. These animals were classified retrospectively as experiencing early embryonic mortality (EEM), late embryonic mortality (LEM) or diagnosed as pregnant, embryonic survival (ES), by presence of embryo with heartbeat at ultrasound exam on D30

On D20, 68.5% of 5-ET cows (*n* = 37/54) had a functional CL (Table 1). On D30, the presence of at least one embryo with heartbeat was confirmed by ultrasound examination in 67.6% of cows that had a functional CL on D20 (*n* = 25/37). Furthermore, cows that had a functional CL on D20 but in which a viable embryo was not detected on D30, the CL remained functional on D30, with the exception of one iP4-supplemented cow that did not have a functional CL on D30. In 63.6% (*n* = 7/11) of cows experiencing LEM, a discrete amount of liquid in the uterine horn ipsilateral to the ovary containing the functional CL was evident. In pregnant cows, a single viable conceptus was verified in 73.3% (*n* = 11/15) of non-iP4 supplemented cows and in 66.6% (*n* = 6/9) of iP4 supplemented cows. Two non-iP4 supplemented cows had three viable conceptuses, while the remaining five cows had two conceptuses. Overall, as represented in Fig. 4, iP4 supplementation doubled the frequency of EEM (iP4: 44.4% vs. Non-iP4: 22.2%) while it did not affect LEM (iP4: 18.6% vs. Non-iP4: 22.2%). Consequently, proportion of pregnancy in iP4 group was numerically lower than that of non-iP4 group (Table 1). Characteristics of CL area and CLBF between D4 and D14 were not affected by pregnancy status nor to P4 concentrations. Analysis considering the mean P4 concentrations over the diestrus, revealed similar results (Table 2). Thus, there was not a specific profile of CL area, CLBF or P4 concentrations associated with the frequency of animals that underwent EEM, LEM or that became pregnant.

**Table 2:** Mean plasma P4 concentrations and *ISG15* abundance according to reproductive status in beef cows

Variable	iP4^a^			Non-iP4			*P* value		
	Pregnant^b^ (*n* = 10)	EEM (*n* = 12)	LEM (*n* = 5)	Pregnant (*n* = 15)	EEM (*n* = 6)	LEM (*n* = 6)	iP4	Group	iP4× Group
Mean plasma P4 concentrations, ng/mL									
D4, D5 and D7	3.21 ± 0.44	4.01 ± 0.38	2.77 ± 0.59	3.17 ± 0.34	2.56 ± 0.54	2.01 ± 0.54	0.04	0.11	0.22
D14 to D20	7.19 ± 0.70	.	4.89 ± 0.94	6.79 ± 0.58	.	6.76 ± 0.86	0.35	0.15	0.16
Mean *ISG15* abundance in PBMCs									
D14 to D20	5.02 ± 0.58	0.76 ± 0.53	3.42 ± 0.82	3.49 ± 0.47	0.76 ± 0.74	3.78 ± 0.74	0.48	< 0.001	0.37
D20	8.56 ± 1.24	1.27 ± 1.31	5.56 ± 1.76	5.79 ± 1.02	0.72 ± 1.97	6.39 ± 1.61	0.50	< 0.001	0.52

*EEM* = early embryonic mortality; *LEM* = late embryonic mortality

^a^Beef cows detected in estrus (D0) were assigned randomly to receive a single injection of vehicle (Non-iP4) or 150 mg of long acting progesterone (iP4) on D

4 and transfer of 5 in vitro produced blastocysts on D7. Conceptus signaling was monitored by abundance of *ISG15* in peripheral blood mononuclear cells (PBMCs) isolated on D14, D16, D18 and D20. Blood samples were collected on D4, D5 and D7 and from D14 to D20, every d, for P4 assay

^b^Cows were classified retrospectively as experiencing early embryonic mortality (EEM), late embryonic mortality (LEM) or diagnosed as pregnant by presence of at least one conceptus with heartbeat at ultrasound exam on D30. Cows were included in the EEM group when there was no evidence of conceptus presence up to D16 according to absence of *ISG15* mRNA increase between D14 and D20 and/or incidence of luteolysis before D20. Conversely, cows classified as LEM presented a clear increase on *ISG15* abundance, functional CL on D30, but no evidence of viable conceptus (i.e. with heartbeat) on D30. Means of variables over time were considered for analysis including the effect of iP4 supplementation, group and interaction. Results are reported as Least squares means ± SEM

**Fig. 5:**
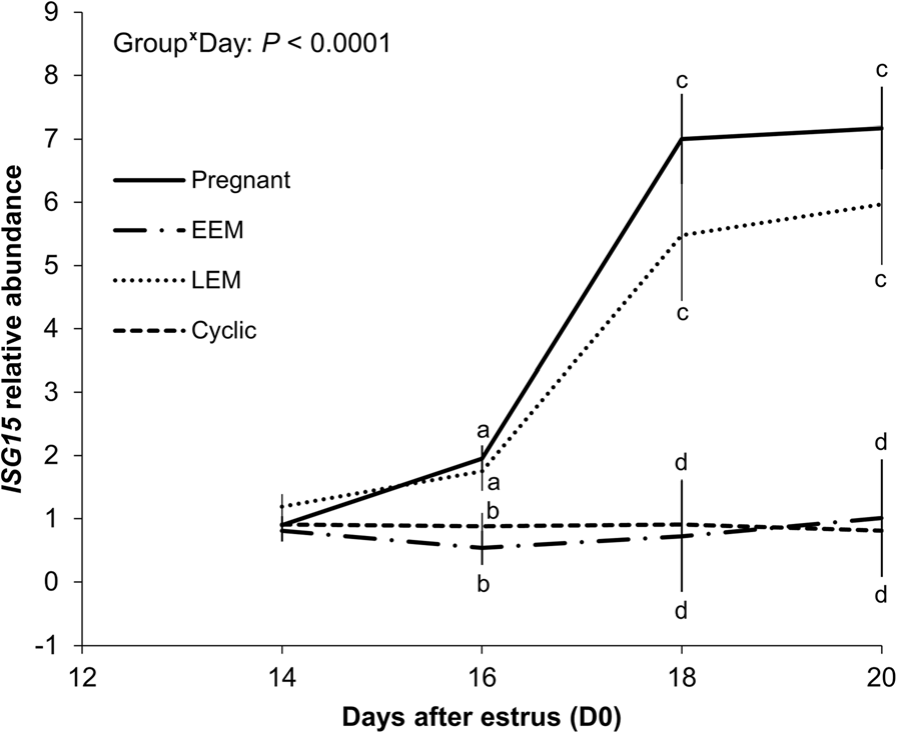
*ISG15* mRNA expression in peripheral blood mononuclear cells according to reproductive status of beef cows. Animals detected in estrus (D0) were assigned randomly to receive a single injection of vehicle (Non-iP4) or 150 mg of long acting progesterone (iP4) on D4 and transfer of none or 5 in vitro produced blastocysts on D7. Cows that received five embryos on D7 were classified retrospectively as experiencing early embryonic mortality (EEM; *n* = 18), late embryonic mortality (LEM; *n* = 11) or confirmed as pregnant at ultrasound exam on D30 (*n* = 25). Cows receiving no embryos were classified as cyclic (*n* = 25). Data were analyzed by split-plot ANOVA and only the significant effect was reported. Data are represented as Least squares means ± SEM. Values within a day without a common superscript are different between groups at ^a,b^*P* < 0.05 or ^c,d^*P* < 0.0001

The relative abundance of *ISG15* in PBMCs during early pregnancy increased over time in pregnant and LEM cows (day by group interaction; Fig. 5). The *ISG15* abundance over time in cows that underwent EEM remained low and similar to the cyclic group. Embryo signalling was first detectable on D16, but only became evident on D18 and D20 (Fig. 5). Consistently, mean *ISG15* abundance from D14 to D20 for pregnant and LEM group was similar and they were 3.49- and 2.84- fold greater than EEM group, respectively (Table 2).

**Fig. 6:**
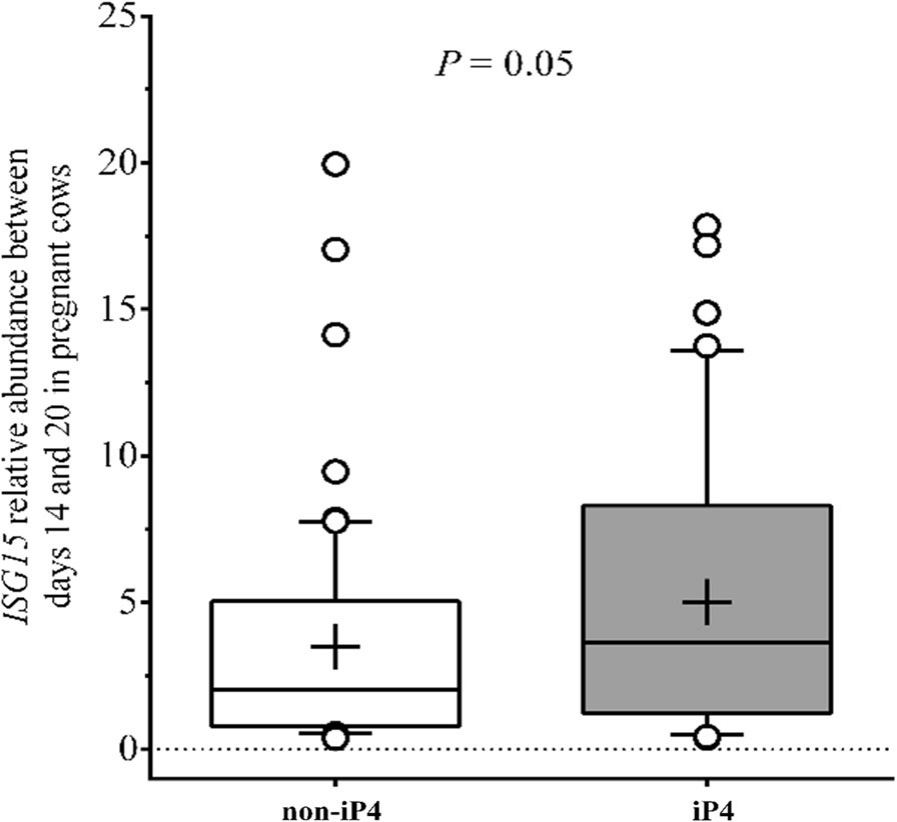
Effect of iP4 supplementation on *ISG15* mRNA expression in PBMCs of beef cows. Animals detected in estrus (D0) were assigned randomly to receive a single injection of vehicle (Non-iP4) or 150 mg of long acting progesterone (iP4) on D4 and transfer of 5 in vitro produced blastocysts on D7. Conceptus signaling in cows diagnosed as pregnant at ultrasound exam on D30 (iP4; *n* = 10 and Non-iP4; *n* = 15) was monitored by abundance of *ISG15* in peripheral blood mononuclear cells (PBMCs) isolated on D14, D16, D18 and D20. Data were analyzed by split-plot ANOVA and only the significant effect was reported. Data are represented as Box-plot using least squares means ± SEM over the time. The average abundance of *ISG15* (between D14 and D20) was greater in iP4 supplemented cows than those Non-iP4 supplemented (mean is represented by the cross in the graphic)

Thirty six out of 37 5-ET cows having a functional CL on D20 also presented an upregulation on *ISG15* abundance over time and a functional CL on D30. The exception was a cow from the iP4 group from which the relative abundance of *ISG15* on D16 (0.97-fold) and D20 (0.50-fold) was essentially unchanged. When only data from pregnant cows were analysed to evaluate the effect of iP4 supplementation on *ISG15* expression, abundance (between D14 and D20) was 1.44-fold greater (*P* = 0.05) in group iP4 compared to the non-iP4 (Fig. 6).

## Discussion

In cattle, early embryonic mortality can be attributed to an incompetent embryo, a non-receptive reproductive tract (i.e., oviduct and/or uterus), a combination of both or a lack of compatibility between the embryo and the uterus. Here, we transferred multiple D7 blastocysts, aiming to minimize pregnancy failures caused by the potential, random developmental inability of a single embryo. Furthermore, embryonic competence was tested in the presence of supplemental P4 (iP4), that was predicted to stimulate conceptus growth and, consequently, a strong antiluteolytic signaling. Our overall expectation was that the proportion of pregnant recipients would be elevated (i.e., > 80%), both on D20 and D30. Despite the greater antiluteolytic activity in iP4-treated cows, indicated by the increased *ISG15* expression in PBMCs, iP4 failed to prevent EEM and LEM and did not improve embryo retention or pregnancy. These effects occurred despite of the absence of iP4-induced short CL lifespan. Our data provided the following novel insights into early pregnancy biology: 1) EEM in non-iP4 cows indicated that a non-receptive uterus may prevent embryo development and signaling; 2) excessive EEM in iP4-treated cows indicated that antiluteolytic signaling was even further disrupted after iP4-priming, and this was not related to early onset of luteolysis; 3) LEM occurred despite successful antiluteolytic signaling during maternal recognition of pregnancy, and was not influenced by the iP4 treatment; 4) even when a given recipient showed ability to retain a pregnancy up to D30, the majority of blastocysts transferred were not able to survive to that stage. This indicates that there may not be a unique set of uterine characteristics that guarantees embryo development.

Contrary to our expectation, frequency of EEM (Non-iP4 + 5-ET: 22.2%) was not substantially reduced by transfer of multiple, in vitro-produced blastocysts and it approached the incidence of EEM (30%) currently described in the literature in cattle [1–3]. This supports the notion that embryo mortality was caused by a functionally incompetent uterus, that was unable to support embryo development at the time of embryo transfer. Results from iP4 + 5-ET group corroborated this concept. Supplementation with iP4 doubled EEM frequency (44.4%), suggesting that it was detrimental, rather than beneficial, for embryonic survival. This adverse result occurred in spite of the P4-stimulated increase of relative *ISG15* abundance in PBMCs (Fig. 6). Such P4 effects indicated that, at least in a proportion of animals, supplemental P4 stimulated conceptus elongation, amplifying intrauterine IFNT signalling. Accordingly, Matsuyama et al. [35] showed a positive relationship between increasing amounts of IFNT infused into the uterus of cows and abundance of *ISG15* mRNA in PBMCs. The effects of supplemental iP4 were before embryo transfer. Indeed, iP4 increased plasma P4 concentrations on D5 (2.7 ng/mL increase), but not on D7 (day of embryo transfer). This supports the notion that P4 actions changed the uterine milieu to affect embryonic development, as reported previously [11, 12]. The effects of iP4 were probably through advancing the expected temporal changes that occur in the endometrial transcriptome along diestrus [36]. Such changes both benefitted (i.e., stimulated *ISG15* abundance) and impaired (greater EEM) embryo survival, but reasons for such contrasting outcomes to the same stimulus are unknown currently. We and others reported previously that early elevation of P4 concentrations during diestrus have increased the incidence of early luteolysis [11, 25, 27, 37]. However, in the present study, iP4 treatment did not reduce luteal lifespan of any animal. Collectively, we propose that functionally incompetent uteri hindered conceptus development.

Frequency of LEM was not affected by iP4 treatment (iP4: 18.5% and Non-IP4: 22.2%). Our results were compatible with the incidence of LEM reported for dairy cows submitted to AI (~ 20%) [1, 38] or to crossbred recipient heifers that received one or two in vitro produced embryos (14%) [39]. Incidence of late embryonic or fetal mortality (after d 28) varies between 8% and 11% for beef cows submitted to AI [2, 40] or crossbred recipient heifers that received one in vitro produced embryo [18, 41]. Causes of LEM can be related to the pre-implantation development phase, such as insufficient growth. Indeed, IFNT acts on the endometrium regulating genes that may be important for implantation and placentation [5, 42, 43]. In a previous study, expression of *ISG15* in PBMCs of cows experiencing LEM after ET was less than that of cows that remained pregnant [35], suggesting that reduced or delayed IFNT secretion is associated with conceptus death that occurs after maternal recognition of pregnancy. In this sense, we anticipated that LEM incidence would be attenuated in the iP4 group due amplification of IFNT signaling. However, *ISG15* abundance was similar between cows experiencing LEM and those that remained pregnant (Fig. 4; Table 2), regardless of iP4 supplementation. Collectively, these results showed that even when the conceptus is able to release IFNT, pregnancy is not necessarily maintained.

The timing of increase of *ISG15* mRNA abundance was compatible with a previous study using AI [29], indicating that neither transfer of multiple blastocysts nor supplemental P4 disrupted the normal timing of IFNT production and sensing by PBMCs, that started around D16 [8]. However, transfer of multiple blastocysts induced unexpectedly earlier luteal demise. Apparently, multiple-conceptuses generated signals to the endometrium that stimulated, rather than inhibited, the luteolytic cascade, in cows experiencing EEM. In both sheep and cattle, the conceptuses and endometrium synthesize a variety of prostaglandins (PGs) during early pregnancy, such as PGE2 and PGF2α, [44, 45]. Indeed, the endometrium produces substantially more PGs during early pregnancy than during the estrous cycle, although release is not pulsatile [46, 47]. Accordingly, prostaglandin synthase two (PTGS2) a key enzyme in PG synthesis, was upregulated in the endometrium of sheep and cows collected at various days of early pregnancy (D10 to D24) [45, 48, 49]. In addition, PTGS2 was induced in the endometrium by IFNT stimulus [49, 50] and by P4 treatment in ovariectomized ewes [45]. Thus, uterine exposure to multiple-embryos may have enhanced PTGS2 expression, stimulating the luteolytic process in cows experiencing EEM. Furthermore, intraluminal uterine concentrations of PGs from embryonic and endometrial origins were probably enhanced in such cows. Despite the clear importance of PGs for embryonic development and maintenance of pregnancy in ruminants [46, 47, 50], PGF2α can also impair early embryonic development [51] and pregnancy rates [52] in cattle. In this regard, the excessive production of PGs and embryonic oversignaling may be deleterious to embryonic survival. Therefore, the iP4 supplementation may have potentiated the side-effects of transferring multiple blastocysts, hindering embryonic survival. This also suggests that exacerbated embryonic signaling is not capable of overcoming inherent uterine deficiencies.

Finally, in this study the pregnancy per ET (iP4: 37.0% and Non-iP4: 55.6%) was compatible with previous reports (37.4% to 56%) for in vitro produced embryos [18, 41, 53], restating the absence of positive effect of the transfer of multiple blastocysts and P4 supplementation on reducing pregnancy losses. It was noteworthy that the majority of cows diagnosed as pregnant (iP4: 66.6% and Non-iP4: 73.3%) presented only a single viable embryo at pregnancy diagnosis on D30, even after the transfer of multiple high-grade, in vitro D7 produced blastocysts. These data suggest that embryonic competence to establish a gestation in a receptive uterine environment was variable. A complementary interpretation is that survivability may be compromised by exacerbated embryonic signaling resulting on excessive production of substances that are toxic to the embryo, such as PGs.

## Conclusion

In conclusion, a substantial number of cows had early (31%) and late (20%) embryonic mortality even after transferring five high-grade, in vitro D7 produced blastocysts. This argues that mortality was due to poor uterine receptivity, that could not be reversed by a single injection of 150 mg of long acting P4 on D4 post-estrus (~D3 post-ovulation). Receptive uteri that responded positively to iP4 resulted in improved conceptus signaling during the maternal recognition of pregnancy window, evidenced by the elevated abundance of *ISG15* in PBMCs of pregnant cows. Further, luteal lifespan was not shortened by iP4 supplementation, demonstrating that iP4-induced earlier luteolysis was not the cause of embryo mortality. Collectively, our results indicated that deficiencies on uterine environment are a main cause of embryonic losses in cattle. Potentiating embryonic signaling through supplementing P4 and transferring multiple in vitro-produced blastocysts was not capable to overcome uterine dysfunction. Finally, we observed that only specific embryos within a cohort exposed to the same uterine environment succeeded to D30. Thus, a given uterine environment is not necessarily adequate to all embryos. Compatibility between embryos and uteri warrants further investigation.

## Abbreviations

AI: artificial insemination
ANOVA: analysis of variance
CL: corpus luteum
CLBF: color blood flow
CV: coefficient of variation
EEM: early embryonic mortality
ET: embryo transfer
INFT: interferon-tau
iP4: injectable long acting progesterone
ISG15: interferon-stimulated gene 15
LEM: late embryonic mortality
mRNA: messenger ribonucleic acid
P4: progesterone
PBMCs: peripheral blood mononuclear cells
PGF2α: prostaglandin F2α
SEM: standard error of the mean
